# The accuracy of using vergence formula to screen myopia in children: a cross-sectional study

**DOI:** 10.3389/fmed.2023.1233080

**Published:** 2023-10-11

**Authors:** Zhen Yi, Chang Hong, Huang Haikuo, Wang Xinxin

**Affiliations:** ^1^National Engineering Research Center for Ophthalmology, Beijing Institute of Ophthalmology, Beijing Tongren Eye Center, Engineering Research Center of the Ministry of Education for Ophthalmic Diagnosis and Treatment Equipment and Materials, Beijing Tongren Hospital, Capital Medical University, Beijing, China; ^2^Beijing He Eye Specialist Hospital, Beijing, China; ^3^Beijing Minghao Technology Development Company, Beijing, China

**Keywords:** myopia – epidemiology, diagnosis, vergence formula, spherical equivalent, axial length, axial length/corneal radius ratio

## Abstract

**Objective:**

To evaluate the accuracy of using the vergence formula to screen myopia in children and adolescents.

**Methods:**

This was a cross-sectional study conducted between December 2022 and May 2023 at the ophthalmology clinic of Beijing Tongren Hospital. A total of 336 children aged 6 to 12 years with refractive errors were selected according to the inclusion criteria. Biometric measurements, including axial length, corneal thickness, anterior chamber depth, corneal curvature, and lens thickness, were obtained using a biometer. The Calculated spherical equivalent (SE) was then calculated using the vergence formula. Cycloplegic refraction was performed after paralysis of the ciliary muscle, and the subjective SE was recorded. A diagnosis of myopia was made if the subjective SE was ≤ −0.50 diopters.

**Results:**

The AL/CR, subjective SE, and calculated SE were not normally distributed (*p* < 0.05). The AL/CR value was 3.08 (2.81, 3.27), the SE was −1.60 D (−6.00 D, 3.75 D), and the calculated SE was −1.42 D (−6.64 D, 5.73 D). There was no significant difference between the calculated SE and the SE (*Z* = −2.899, *p* = 0.004). The AL/CR value was negatively correlated with SE (*r* = −0.687, *p* < 0.01), and the calculated SE was positively correlated with SE (*r* = 0.827, *p* < 0.01). The area under the ROC curve for predicting myopia using AL/CR and calculated SE was 0.876 and 0.962, respectively, and the difference between the two was significant (*p* < 0.001). The sensitivity of AL/CR was 84.2%, the specificity was 70.6%, the accuracy was 82.1%, and the Youden index was 0.548. The sensitivity of calculated SE was 83.1%, the specificity was 100%, the accuracy was 85.7%, and the Youden index was 0.831.

**Conclusion:**

The vergence formula can be used to evaluate myopia in children and adolescents with relatively high accuracy without cycloplegic refraction.

## Introduction

Screening is an important step in the early diagnosis and intervention of myopia. Currently, common methods for screening myopia include uncorrected visual acuity (UCVA), objective refraction under small pupils, and axial length measurement (1). While UCVA is simple and easy to operate, it has high subjectivity and variability, and cannot accurately and objectively reflect the refractive state of children. The sensitivity and specificity are both low, so it can only be used as a preliminary screening indicator. Cycloplegic refraction has always been the gold standard for diagnosing myopia, but it is not fast and has poor comfort, making it difficult to be accepted by young patients and their families. Objective refraction under small pupils can avoid the use of mydriatic, but due to the strong accommodative ability of children, it is easy to produce accommodation, resulting in high sensitivity but low specificity, which may lead to a high rate of misdiagnosis and cannot accurately reflect the true prevalence of the myopia (2, 3).

Dynamic monitoring of axial length can help detect myopia in a timely manner, and the measurement process is non-invasive, simple, and easy to perform, which can be better accepted by children and their families (4, 5). It is a good objective indicator for evaluating the development of refraction. However, many studies have shown that refractive parameters such as axial length, corneal radius, and lens thickness constantly change, and the balance state of these parameters ultimately determines the refractive state of the eye. A single factor of axial length cannot explain the final change in refractive state well. Some researchers have used the axial length-to-corneal radius (AL/CR) ratio to predict whether myopia will occur, which has shown higher diagnostic value than axial length alone (6, 7). However, AL/CR ratio can only make qualitative predictions about the occurrence of myopia and cannot establish a quantitative relationship with the refractive power after dilation and cannot directly calculate the hyperopia reserve.

The vergence formula has been widely used for calculating the power of artificial lenses (Holladay 1, SRK/T, Hoffer Q, Haigis formula), which is characterized by the introduction of the concept of effective lens position (ELP). Based on this formula, we can calculate the power of the lens needed to be implanted based on the axial length, corneal curvature, anterior chamber depth, refractive errors, and lens position. The author used this formula to establish a new method for calculating refractive errors based on axial length, corneal curvature, anterior chamber depth, and lens thickness data. To test the accuracy of the new method, we conducted a clinical study on young children.

## Methods

### Using the vergence formula for refraction calculation

First, we assume an eye with an axial length (*AL*) and overall refractive power (*P_E_*), corneal refractive power (*P_C_*), refractive index of the aqueous humor (*n*), refractive power of the lens (*P_L_*), lens thickness (*T_L_*), and effective lens position (*ELP*), which is the distance between the principal plane of the lens and the anterior corneal vertex and can be calculated as follows:


$$\begin{array}{l} ELP=\mathrm{corneal}\;\mathrm{thickness}+\mathrm{anterior}\;\mathrm{chamber}\;\mathrm{depth}\\ \;+\;0.5\times \mathrm{lens}\;\mathrm{thickness} \end{array}$$

The vergence formula is U + P = V, where U is the incoming refractive power, *P* is the lens refractive power, and V is the outgoing refractive power, all measured in diopters. Based on this formula, the overall refractive power of the eye can be calculated as follows:


$$P_{E}=\frac{n}{AL-ELP}$$

We can also use this formula to calculate the *P_C_* on the lens plane. If a parallel beam of light passes through the cornea, the focal point will be at *n*/*K*. If we move the cornea to the lens plane, the corneal refractive power will be adjusted as follows:


$$P_{C}=\frac{n}{\frac{n}{K}-ELP}$$

Here, *K* is the corneal curvature measured by a biometer.

Assuming that the refractive power of the lens is proportional to its thickness, we can calculate *P_L_* as follows:


$$P_{L}=A*T_{L}$$

Where *T_L_* is the thickness of the lens measured by a biometer, and *A* is a constant that can be obtained using the following method:


$$P_{E}=P_{C}+P_{L}$$

$$P_{L}=P_{E}-P_{C}$$

$$A=\frac{P_{E}-P_{C}}{T_{L}}$$

By collecting biometric data on patients with different refractive errors, we can obtain *P_E_*, *P_C_*, and *T_L_*. For patients with refractive errors, the *K* value of *P_C_* is adjusted as follows:


$$K_{adj}=K+\frac{1}{\frac{1}{R_{x}}-V}$$

Here, *R_X_* is the spherical power of the meridian, and *V* is the vertex distance, which is usually taken as 12 mm. Finally, we can calculate *A* for a specific population using the following formula:


$$A=\frac{\frac{N}{AL-ELP}-\frac{n}{\frac{n}{K+\frac{1}{\frac{1}{R_{x}}-V}}-ELP}}{T_{L}}$$

In the preliminary study, cycloplegic refraction and axial length data were collected from 105 children of similar age, and the *A* constant for this population was calculated. Assuming that the *A* constant is applicable to other populations, it can be used for calculations. Thus, we can calculate *P_E_*, *P_C_*, and *P_L_* using the measurements of axial length, corneal thickness, corneal curvature, anterior chamber depth, and lens thickness. The refractive error in the meridian plane can be calculated as follows:


$$D=P_{E}-P_{C}-P_{L}$$

We can then calculate the refractive error values *D*_1_ and *D*_2_ for the corresponding meridians *K*_1_ and *K*_2_, respectively, and the calculated spherical equivalent (SE) value can be get using the following formula:


$$D_{SE}=D_{1}+D_{2}*0.5$$

### Subject

A total of 336 myopic children aged 6–12 years old, who were first diagnosed at the ophthalmology outpatient department of Beijing Tongren Hospital between December 2022 and May 2023, were included in this study. Among them, there were 175 boys (52.1%) and 161 girls (47.9%), with an average age of 9.2 ± 2.6 years. The inclusion criteria were able to cooperate with vision, cycloplegic refraction, axial length measurement, slit lamp biomicroscopy, fundus examination, and other relevant ophthalmic examinations; parents provided informed consent and signed the informed consent form. Exclusion criteria were other eye diseases such as strabismus, cataracts, glaucoma, and fundus diseases; a history of surgical trauma or systemic diseases; incomplete examination data. This research was approved by the Human Studies Committee of Beijing Tongren Hospital (Beijing, China) in accordance with the Code of Ethics of the World Medical Association (registration number: ChiCTR2100047074).

Examination procedures: A visual acuity test was performed by an optometrist using a standard logarithmic visual acuity chart. Refraction and corneal curvature were measured under small pupils using a computerized refractometer (KR-8900, Topcon Corporation, Japan) with three measurements and the average recorded. Biometric parameters were measured in both eyes using an optical coherence biometer (OA-2000, Tomey Corporation, Japan) with three measurements and the average recorded. The mydriatic agent tropicamide (American Alcon Inc.) was used twice, 30 min apart, to dilate the pupils of children. An optometrist confirmed that the pupil diameter was greater than 6 mm and performed retinoscopy and subjective refraction to record the SE.

Statistical analysis: This study was a cross-sectional study, and statistical analysis was performed using SPSS 23.0 software. *p* < 0.05 was considered statistically significant. Since the parameters of both eyes were highly correlated, we only analyzed data from the right eye. Qualitative data were expressed as frequency (percentage), and quantitative data were tested for normality. If the data were not normally distributed, the median (maximum value, minimum value) was used. The correlation between AL/CR, calculated SE and SE was analyzed by rank correlation analysis. An SE ≤ −0.50 D was used as the gold standard for diagnosing myopia, and an SE ≤ −0.50 D was used as the positive threshold for myopia diagnosis. AL/CR >3 was considered suspicious for myopia, and ≤ 3 was considered non-myopia. The true positive, false positive, false negative, and true negative values of AL/CR and calculated SE in diagnosing myopia in children were calculated, and sensitivity, specificity, accuracy, misdiagnosis rate, missed diagnosis rate, positive predictive value, negative predictive value, positive likelihood ratio, negative likelihood ratio, and Youden index were further calculated.

## Results

### Refractive parameters according to demographic characteristics

**Figure 1:**
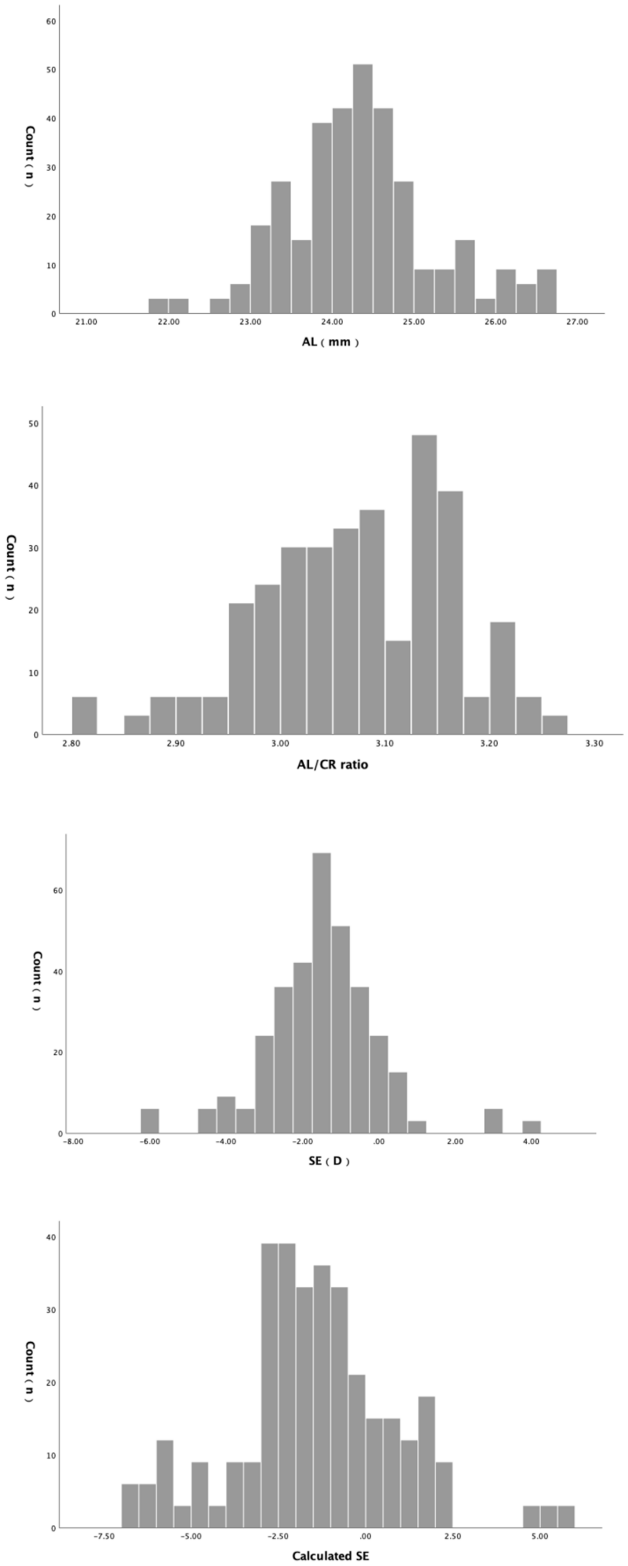
The histogram of refractive parameters among participants.

The histogram of refractive parameters among participants in this study is shown in Figure 1. A total of 285 (84.8%) participants suffers from myopia. After a normality test, AL, AL/CR, calculated SE and SE were found not to follow a normal distribution. AL was 24.26 (21.88, 26.70), and AL/CR was 3.08 (2.81, 3.27). SE was −1.60D (−6.00D, 3.75D), calculated SE was −1.42D (−6.64D, 5.73D), and the difference was 0.18 (−2.80, 2.53). Wilcoxon rank-sum test showed that the difference between the two was significant (*Z* = −2.899, *p* = 0.004).

### Correction analysis between SE, AL/CR ratio, and AL

**Figure 2:**
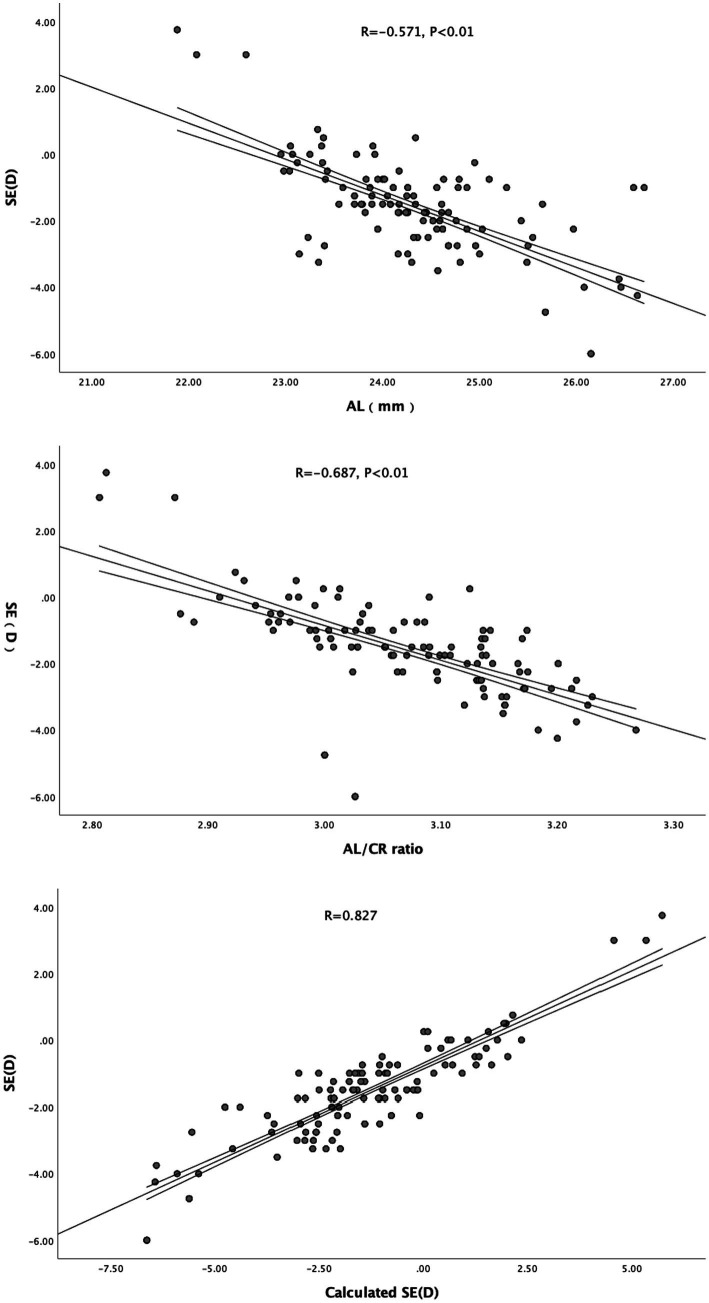
The scatter plots between AL, AL/CR ratio, calculated SE and SE.

The scatter plots between AL, AL/CR ratio, calculated SE and SE are shown in Figure 2. AL is negatively correlated with SE (*r* = −0.571, *p* < 0.01), AL/CR is negatively correlated with SE (*r* = −0.687, *p* < 0.01), and calculated SE is positively correlated with SE (*r* = 0.827, *p* < 0.01).

### Accuracy of AL/CR ratio and calculated SE for myopia assessment

**Figure 3:**
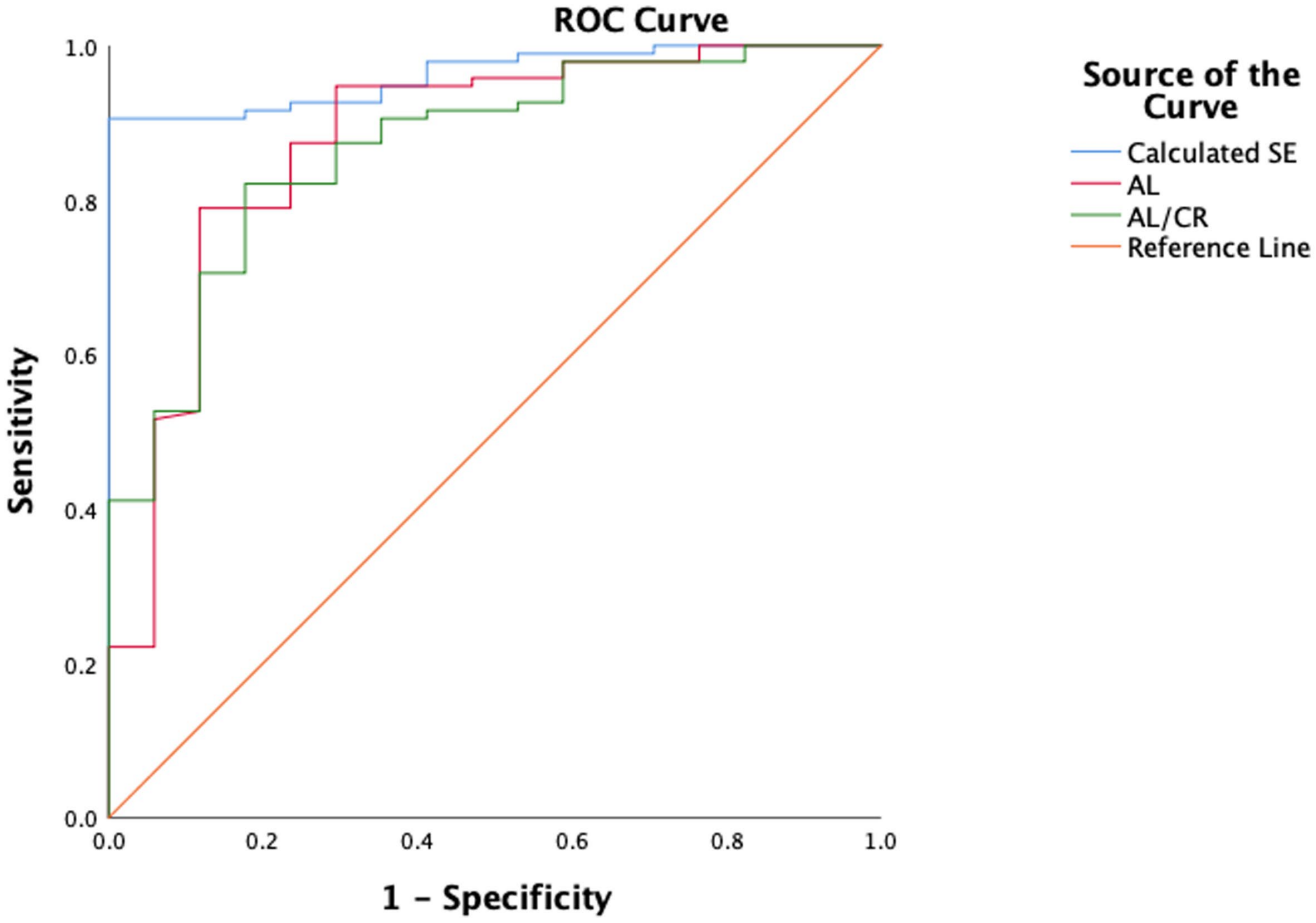
The comparison of the accuracy of calculated SE, AL and AL/CR ratio for myopia assessment. AUC, area under the curve.

Taking cycloplegic refraction SE ≤ −0.50 D as the gold standard for diagnosis of myopia, the accuracy of AL/CR ratio and calculated SE for myopia assessment were analyzed. The ROC curves were drawn using AL, AL/CR ratio and calculated SE as the index for myopia assessment, and the AUC of ROC curves was 0.876 (95% confidence interval [CI]: 0.818–0.934), 0.867 (95% CI: 0.816–0.917) and 0.962 (95% CI: 0.944–0.980) (Figure 3). The area under the ROC curve of Calculated SE was significantly higher than that of AL/CR (*p* < 0.001) and AL (*p* < 0.001), and there was no significant difference in the area under the ROC curve between AL and AL/CR (*p* = 0.813).

The number of cases judged as myopia or not based on SE, AL/CR, and Calculated SE are shown in Tables 1, 2. The sensitivity, specificity, accuracy, and Youden index of AL/CR were 84.2, 70.6, 82.1%, and 0.548, respectively. The false-positive rate and missed diagnosis rate were 29.4 and 15.8%, respectively. The positive predictive value was 94.1%, the negative predictive value was 44.4%, and the positive and negative likelihood ratios were 2.86 and 0.22, respectively. The sensitivity, specificity, accuracy, and Youden index of Calculated SE were 83.1, 100, 85.7%, and 0.831, respectively. The false-positive rate and missed diagnosis rate were 0 and 16.9%, respectively. The positive predictive value was 100%, the negative predictive value was 51.5%, and the positive and negative likelihood ratios were infinite and 0.17, respectively.

**Table 1:** Frequency statistics of myopia diagnosis using SE and Calculated SE.

		SE		Total
		Emmetropia	Myopia	
Calculated SE	Emmetropia	51	48	99
	Myopia	0	237	237
	Total	51	285	336

**Table 2:** Frequency statistics of myopia diagnosis using SE and AL/CR.

		SE		Total
		Emmetropia	Myopia	
AL/CR	Emmetropia	36	45	81
	Myopia	15	240	255
	Total	51	285	336

## Discussion

In this study, we found that the correlation between SE and calculated SE is stronger than that between SE and AL/CR in children. The accuracy of the calculated SE for myopia assessment was higher than that of the AL/CR ratio.

In recent years, the prevalence of myopia has been constantly increasing, especially in East Asia, where 80 to 90% of 18-year-old adolescents are myopic, and 10 to 20% of the population have high myopia (8). Based on current trends, it is estimated that by 2050, there will be 4.758 billion myopia patients and 938 million high myopia patients worldwide (9). Preventing the onset and progression of myopia is crucial, as it poses a persistent threat to quality of life, and high myopia may further complicate many vision-damaging diseases, including myopic macular degeneration and glaucoma (10, 11). Myopia screening helps identify high-risk children for more timely and effective intervention, slowing the onset or progression of myopia, improving visual performance and quality of life (10, 12).

Baseline refraction and ocular biometry have long been considered risk factors for the onset and development of myopia. Zadnik et al. (13) were the first to use refraction and ocular biometry parameters to screen myopia, and the results showed that the best single indicator for screening myopia was the cycloplegic refraction spherical equivalent (SE), with an AUC of 0.880, sensitivity of 86.7%, and specificity of 73.3%. When corneal curvature, lens power, and axial length (AL) were added to the model, the AUC increased slightly to 0.893. Zadnik et al. (14) found that increasing the number of predictive factors from only baseline SE to baseline SE, parental myopia, AL, corneal curvature, lens power, accommodative convergence-to-accommodation ratio (AC/A ratio), horizontal/vertical astigmatism, and visual acuity (VA) only increased the AUC by 0.01 to 0.02. Ma et al. (15) also found that single baseline SE could provide effective prediction for future myopia. In their study of 1856 students from Shanghai, China, the AUC for predicting 4 years myopia (SE ≤ −0.5D in the amblyopic eye) was 0.585, 0.740, and 0.839 for baseline AL, AL/CR, and SE, respectively. Compared to using baseline SE alone, combining baseline SE, AL/CR, age, sex, and parental myopia only increased the AUC by 0.022. As children have strong accommodative ability and are prone to near work, the SE obtained after ciliary muscle paralysis remains the gold standard for screening myopia. However, due to the long time required for cycloplegic refraction and the potential risk of medication, it is difficult to use in large-scale screening in school settings.

Most of the myopia in children is axial myopia, which occurs when the AL exceeds the normal range and cannot be matched with other refractive components, resulting in myopia. Previous studies have shown that the changes in ocular biometric parameters before and after mydriasis are smaller than diopters (16–18). Tao et al. (19) found that even in young children who use atropine for mydriasis, there is no significant change in ocular biometric parameters. The invention of the optical coherence tomography (OCT) biometer has enabled non-invasive and easy measurement of ocular biometric parameters, which can be better accepted by subjects and their families and serve as a good objective indicator for evaluating refractive status.

During the process of emmetropization, the average corneal curvature radius increases with the growth of the axial length to maintain emmetropia. However, when the increase in average corneal curvature radius is insufficient to compensate for the excessive growth of the axial length, myopia occurs. Goss et al. (20) found that an axial ratio of 3 is the critical point for the limit of compensatory increase in average corneal curvature radius, and children with an AL/CR ratio > 3 are prone to myopia. Mu et al. (21) showed that the area under the ROC curve for screening myopia in school-age children using the AL/CR ratio was 0.937 (95% confidence interval: 0.878–0.996), with specificity, sensitivity, Youden’s index, positive predictive value, and negative predictive value of 0.703, 0.913, 0.622, 0.956, and 0.771, respectively. Liu et al. (22) found that the AL/CR ratio can also be used as an indicator for identifying pre-school children who are about to develop myopia. The use of the AL/CR ratio to predict the onset and progression of myopia has clinical significance when children cannot or do not want to undergo cycloplegic refraction testing. However, the AL/CR ratio can only qualitatively estimate the presence or progression of myopia and cannot be directly converted to diopters, which makes it difficult to quantitatively evaluate the risk of myopia onset and progression.

According to the vergence formula, we can calculate the SE by knowing the axial length, corneal curvature, and lens power. Although measurements of axial length and corneal curvature have become routine in clinical practice, direct measurement of lens power is still not possible. Bennett (23) developed a formula for calculating lens power using the Gullstrand-Emsley model eye, which showed good consistency with phakometry measurements. However, the formula requires measurement of the curvature radius of the lens surfaces, which current biometers cannot achieve, making it unsuitable for large-scale studies or clinical practice (24).

The main innovation of this study is the proposal that lens power is a function related to lens thickness, which simplifies the calculation process. This hypothesis is based on the phenomenon that lens power decreases when the lens becomes thinner during accommodation relaxation and when the lens becomes thicker during accommodation tension. Results show that the difference in SE values calculated using this method and those obtained after cycloplegia is lower (0.18D), and the two are highly correlated, with a correlation coefficient higher than that of axial length and the AL/CR ratio. The accuracy of the qualitative diagnosis of myopia and the area under the ROC curve were also higher than those of AL and the AL/CR ratio. This indicates that the calculated SE using the simplified formula can meet the needs of screening for myopia and preliminarily verifies the accuracy of the formula in assessing myopia in adolescents.

Although the median values of the difference between SE and calculated SE are not large, there is a large fluctuation range (−2.80, 2.53), which is similar to the results of Rozema et al. (25) who used the Bennett formula to calculate lens power (0.37 ± 1.56D). The authors analyze that this may be related to the simple conversion between lens thickness and lens power using a single constant A, and in the next step of research, they will attempt to reduce the fluctuation of the predicted results using regression analysis, machine learning, and other methods to speed up the convergence of the model, to more accurately quantify the assessment of myopia in adolescents.

In summary, this study established a new method for calculating post-cycloplegic refractive error based on pre-cycloplegia ocular biometric parameters using the formula for spherical equivalent. The accuracy of this method in qualitatively diagnosing myopia in children is higher than that of the AL/CR ratio and can reduce the dependence on cycloplegic refraction during myopia screening.

## Data availability statement

The original contributions presented in the study are included in the article/supplementary material, further inquiries can be directed to the corresponding author.

## Ethics statement

The studies involving humans were approved by Human Studies Committee of Beijing Tongren Hospital (Beijing, China). The studies were conducted in accordance with the local legislation and institutional requirements. Written informed consent for participation in this study was provided by the participants’ legal guardians/next of kin.

## Author contributions

ZY, CH, HH, and WX contributed to the study conception and design. Material preparation, data collection and analysis were performed by ZY and CH. The first draft of the manuscript was written by ZY. ZY, CH, HH, and WX commented on previous versions of the manuscript. WX and HH: critical revision of the manuscript for important intellectual content. All authors contributed to the article and approved the submitted version.

## References

[1] Jonas JB Ang M Cho P Guggenheim JA He MG Jong M. IMI prevention of myopia and its progression. Invest Ophthalmol Vis Sci. (2021) 62:6. doi: 10.1167/iovs.62.5.6, PMID: 33909032 PMC8083117

[2] Guo R Shi L Xu K Hong D. Clinical evaluation of autorefraction and subjective refraction with and without cycloplegia in Chinese school-aged children: a cross-sectional study. Transl Pediatr. (2022) 11:933–46. doi: 10.21037/tp-22-226, PMID: 35800271 PMC9253959

[3] Wilson LB Melia M Kraker RT VanderVeen DK Hutchinson AK Pineles SL. Accuracy of autorefraction in children: a report by the American Academy of ophthalmology. Ophthalmology. (2020) 127:1259–67. doi: 10.1016/j.ophtha.2020.03.004, PMID: 32317177

[4] Fan Q Wang H Jiang Z. Axial length and its relationship to refractive error in Chinese university students. Cont Lens Anterior Eye. (2022) 45:101470. doi: 10.1016/j.clae.2021.101470, PMID: 34030907

[5] Demir P Baskaran K Theagarayan B Gierow P Sankaridurg P Macedo AF. Refractive error, axial length, environmental and hereditary factors associated with myopia in Swedish children. Clin Exp Optom. (2021) 104:595–601. doi: 10.1080/08164622.2021.1878833, PMID: 33689658

[6] Foo VH Verkicharla PK Ikram MK Chua SY Cai S Tan CS. Axial length/corneal radius of curvature ratio and myopia in 3-year-old children. Transl Vis Sci Technol. (2016) 5:5. doi: 10.1167/tvst.5.1.5, PMID: 26929885 PMC4757459

[7] Vera-Diaz FA Jnawali A Panorgias A Bex PJ Kerber KL. Baseline metrics that may predict future myopia in young children. Ophthalmic Physiol Opt. (2023) 43:466–81. doi: 10.1111/opo.13113, PMID: 36892148 PMC10416753

[8] Wolffsohn JS Calossi A Cho P Gifford K Jones L Jones D. Global trends in myopia management attitudes and strategies in clinical practice − 2019 update. Cont Lens Anterior Eye. (2020) 43:9–17. doi: 10.1016/j.clae.2019.11.002, PMID: 31761738

[9] Holden BA Fricke TR Wilson DA Jong M Naidoo KS Sankaridurg P. Global prevalence of myopia and high myopia and temporal trends from 2000 through 2050. Ophthalmology. (2016) 123:1036–42. doi: 10.1016/j.ophtha.2016.01.006, PMID: 26875007

[10] Ha JJ He M. Preventing myopia in East Asia. Community Eye Health. (2019) 32:13–4. PMID: 31409948 PMC6688404

[11] Raval N Kang JJ Kim YH. A review of pathologic myopia. Eye Contact Lens. (2022) 48:403–9. doi: 10.1097/icl.0000000000000917 36155945

[12] Zhu Z Chen Y Tan Z Xiong R McGuinness MB Müller A. Interventions recommended for myopia prevention and control among children and adolescents in China: a systematic review. Br J Ophthalmol. (2023) 107:160–6. doi: 10.1136/bjophthalmol-2021-319306, PMID: 34844916

[13] Zadnik K Mutti DO Friedman NE Qualley PA Jones LA Qui P. Ocular predictors of the onset of juvenile myopia. Invest Ophthalmol Vis Sci. (1999) 40:1936–43. PMID: 10440246

[14] Zadnik K Sinnott LT Cotter SA Jones-Jordan LA Kleinstein RN Manny RE. Prediction of juvenile-onset myopia. JAMA Ophthalmol. (2015) 133:683–9. doi: 10.1001/jamaophthalmol.2015.0471, PMID: 25837970 PMC4607030

[15] Ma Y Zou H Lin S Xu X Zhao R Lu L. Cohort study with 4-year follow-up of myopia and refractive parameters in primary schoolchildren in Baoshan District, Shanghai. Clin Exp Ophthalmol. (2018) 46:861–72. doi: 10.1111/ceo.13195, PMID: 29577563 PMC6282580

[16] Wang X Dong J Tang M Wang X Wang H Zhang S. Effect of pupil dilation on biometric measurements and intraocular lens power calculations in schoolchildren. PLoS One. (2018) 13:e0203677. doi: 10.1371/journal.pone.0203677, PMID: 30212545 PMC6136745

[17] Balsak S. Effects of pupillary dilation on ocular optical biometry outcomes in pediatric patients. Arq Bras Oftalmol. (2020) 83:289–93. doi: 10.5935/0004-2749.20200041, PMID: 32756786 PMC11826585

[18] Adler G Shahar J Kesner R Rosenfeld E Fischer N Loewenstein A. Effect of pupil size on biometry measurements using the IOLMaster. Am J Ophthalmol. (2015) 159:940–4. doi: 10.1016/j.ajo.2015.01.025, PMID: 25637178

[19] Tao Y Li M Tan J Huang J Cheng X Xie P. Effects of atropine and tropicamide on ocular biological parameters in children: a prospective observational study. BMC Ophthalmol. (2023) 23:96. doi: 10.1186/s12886-023-02840-5, PMID: 36915059 PMC10010000

[20] Goss DA Van Veen HG Rainey BB Feng B. Ocular components measured by keratometry, phakometry, and ultrasonography in emmetropic and myopic optometry students. Optom Vis Sci. (1997) 74:489–95. doi: 10.1097/00006324-199707000-00015, PMID: 9293515

[21] Mu J Zeng D Fan J Liu M Zhong H Shuai X. The accuracy of the axial length and axial length/corneal radius ratio for myopia assessment among Chinese children. Front Pediatr. (2022) 10:859944. doi: 10.3389/fped.2022.859944, PMID: 36147807 PMC9488664

[22] Liu L Li R Huang D Zhu H Wang Y Zhao X. Prediction of premyopia and myopia in Chinese preschool children: a longitudinal cohort. BMC Ophthalmol. (2021) 21:283. doi: 10.1186/s12886-021-02045-8, PMID: 34289821 PMC8296532

[23] Bennett AG. A method of determining the equivalent powers of the eye and its crystalline lens without resort to phakometry. Ophthalmic Physiol Opt. (1988) 8:53–9. doi: 10.1016/0275-5408(88)90089-0, PMID: 3047630

[24] Rosales P Dubbelman M Marcos S van der Heijde R. Crystalline lens radii of curvature from Purkinje and Scheimpflug imaging. J Vis. (2006) 6:5–67. doi: 10.1167/6.10.5, PMID: 17132077

[25] Rozema JJ Atchison DA Tassignon MJ. Comparing methods to estimate the human lens power. Invest Ophthalmol Vis Sci. (2011) 52:7937–42. doi: 10.1167/iovs.11-7899 21873657

